# Salvianolic Acid A Improves Rat Kidney Injury by Regulating MAPKs and TGF-β1/Smads Signaling Pathways

**DOI:** 10.3390/molecules28083630

**Published:** 2023-04-21

**Authors:** Hai-Yang Diao, Wei Zhu, Jie Liu, Sheng Yin, Jin-Hui Wang, Chun-Li Li

**Affiliations:** 1 Department of Pharmacology, Shenyang Pharmaceutical University, Shenyang 110016, China; 2 Department of Traditional Chinese Medicine, Shenyang Pharmaceutical University, Shenyang 110016, China; 3 Department of Pharmacy, Harbin Medical University, Harbin 150081, China

**Keywords:** salvianolic acid A, kidney disease, MAPKs signaling pathway, TGF-β1/Smads signaling pathway

## Abstract

Salvianolic acid A (SAA) is one of the major components in *Salvia miltiorrhiza Bge.*, with various pharmacological activities, and is likely to be a promising agent for the treatment of kidney diseases. The purpose of this study was to explore the protective effect and mechanisms of SAA on kidney disease. In this study, the improvement effects of SAA (10, 20, 40 mg/kg, i.g.) on kidney injury rats were investigated by detecting the levels of KIM-1, NGAL in serum and UP in the urine of AKI model rats established with gentamicin, as well as the levels of SCr and UREA in serum and IL-6, IL-12, MDA and T-SOD in the kidneys of CKD model rats established with 5/6 nephrectomy. HE and Masson staining were used to observe the histopathological changes in the kidney. Network pharmacology and Western blotting were used to explore the mechanism of SAA in improving kidney injury. The results showed that SAA improved kidney function in kidney injury rats by reducing the kidney index and pathological injury by HE and Masson staining, reducing the levels of KIM-1, NGAL and UP in AKI rats and UREA, SCr and UP in CKD rats, as well as exerting anti-inflammatory and anti-oxidative stress effects by inhibiting the release of IL-6 and IL-12, reducing MDA and increasing T-SOD. Western blotting results showed that SAA significantly reduced the phosphorylation levels of ERK1/2, p38, JNK and smad2/3, and the expression of TLR-4 and smad7. In conclusion, SAA plays a significant role in improving kidney injury in rats and the mechanism may be achieved by regulating the MAPKs and TGF-β1/smads signaling pathways.

## 1. Introduction

Kidney disease, as a serious health concern, has affected more than 750 million people worldwide [1]. According to the urgency of onset, kidney diseases can be divided into acute kidney injury (AKI) and chronic kidney disease (CKD). The population prevalence of CKD exceeds 10%, and in high-risk subpopulations, exceeds 50% [2]. As the population ages, the incidence of both AKI and CKD increases gradually [3,4].

AKI is a clinical syndrome characterized by rapid loss of kidney excretory function, with abnormal blood, urine, tissue and other clinical indicators [5]. AKI syndrome can develop as a consequence of different pathological conditions, including cardiac surgery, drugs, toxins, end-stage kidney disease, etc. [6]. Exogenous drugs or poisons were used to stimulate AKI through their side effects or poisoning effects, in which AKI can be induced in rats after the injection of gentamicin (40–200 mg/kg) for 4–10 days [7,8,9]. Kidney injury molecule-1 (KIM-1) and neutrophil gelatinase-associated lipocalin (NGAL) have been widely used as early diagnostic markers for AKI, indicating proximal tubular dysfunction and glomerular injury [10,11,12].

AKI is a risk factor for progression to CKD, which may be caused by microcirculation disturbance, chronic inflammatory cell infiltration, nephron loss and kidney fibrosis due to abnormal repairs of the kidney [13,14]. Meanwhile, the end-stage of CKD requires dialysis and even kidney transplantation, which seriously affect the life and health of people with kidney injury [15]. The 5/6 nephrectomized model is a classic CKD model, and studies have proved that after the 5/6 Nx, remaining nephrons are overworked, resulting in kidney tubule atrophy, glomerular sclerosis, kidney interstitial inflammatory cell infiltration and other pathological changes, which can be identified by the changes in serum creatinine (SCr), urea (UREA) and urine protein (UP) levels [16,17,18,19].

**Figure 1:**
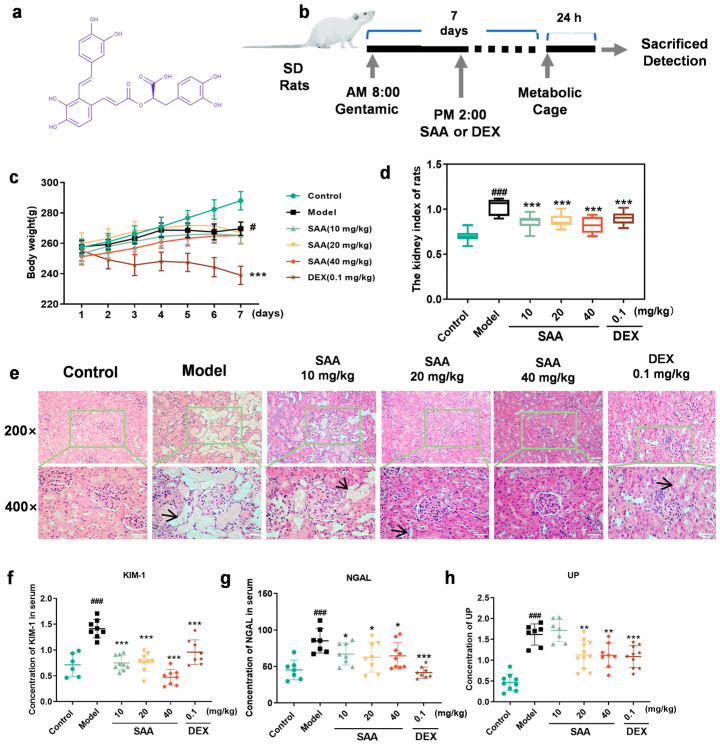
SAA suppressed kidney damage in AKI rats. (**a**) Characterization of SAA. (**b**) Flow chart for preparation of AKI model in SD rats. (**c**) The effect of SAA on the body weight. (**d**) The effect of SAA on the kidney index of rats. (All data are shown as mean ± SEM, *n* = 10–12. # *p* < 0.05, ### *p* < 0.001 compared with Control group; *** *p* < 0.001 compared with Model group.) (**e**) The effects of SAA on histopathological changes in rats. Kidney histopathological changes were observed by H&E staining (*n* = 3, magnification: 200×, 400×, scale bar = 100 μm, 50 μm), black arrow indicates kidney tubular dilatations. (**f**,**g**) Effect of SAA on serum (**f**) KIM-1 and (**g**) NGAL in rats with AKI. (**h**) The effect of SAA on the content of UP. (Green circle represents Control. Black square represents Model. Light green triangle represents 10 mg/kg SAA. Yellow inverted triangle represents 20 mg/kg SAA. The red diamond represents 40 mg/kg SAA. Brown asterisk represents DEX. All data are shown as mean ± SEM, *n* = 6–10. ### *p* < 0.001 compared with Control group; * *p* < 0.05, ** *p* < 0.01, *** *p* < 0.001 compared with Model group.)

Salvianolic acid A (SAA), as shown in Figure 1a, is one of the major water-soluble components in *Salvia miltiorrhiza Bge.* SAA has been reported to possess a wide range of pharmacological activities, including anti-inflammatory, anti-cancer, anti-oxidative stress, anti-fibrosis, etc. [20,21,22,23]. Recent studies have shown that SAA is effective in improving kidney injury and fibrosis. It has been reported that SAA exerts anti-inflammatory and anti-oxidative stress effects by activating the Akt/GSK-3β/Nrf2 signaling pathway and inhibiting the NF-κB signaling pathway in 5/6Nx rats [24]. Meanwhile, in LPS-induced AKI mouse, SAA improves kidney injury by inhibiting the activation of the TLR4/MyD88 signaling pathway and thus reducing the release of inflammatory factors [25]. As a multi-target agent, SAA shows its potential in the treatment of kidney diseases [26,27,28]. According to previous studies of SAA, we speculated that SAA could play an improvement role in AKI and CKD rats.

## 2. Results

### 2.1. SAA Improved Gentamicin-Induced Acute Kidney Injury

The experimental design for the effects of SAA on AKI is shown in Figure 1b, and the effects of body weight and the kidney index are shown in Figure 1c,d. Compared with the Control group, the body weight of the Model group rats decreased significantly on day 7 after the injection of gentamicin (100 mg/kg), and the kidney index of Model group rats increased significantly; compared with the Model group, the kidney index of SAA (10, 20, 40 mg/kg, i.g.) group rats decreased significantly, SAA had no significant effect on the body weight, while the DEX (0.1 mg/kg, i.g.) group decreased significantly, which may be caused by the bitter taste of DEX and the poor compliance of rats.

To further confirm the protective effect of SAA on AKI rats, the results of HE staining were shown in Figure 1e. Compared with the Control group, the kidneys of rats in the Model group showed pathological changes such as cystic and tubular dilatations. Compared with the Model group, SAA significantly improved kidney pathological damage in a dose-dependent manner. The above results indicate that SAA improves kidney injury in gentamicin-induced AKI rats.

### 2.2. SAA Reduced KIM-1, NGAL and UP Levels in AKI Rats

The levels of KIM-1 and NGAL were detected by ELISA, as shown in Figure 1f,g. Compared with the Control group, the levels of KIM-1 and NGAL in serum were increased significantly in the Model group. Compared with the Model group, the levels of KIM-1 and NGAL were significantly decreased in the SAA and the DEX groups. The quantitative result of UP is shown in Figure 1h; compared with the Control group, the content of UP in the Model group increased significantly, and compared with the Model group, the contents of UP in the SAA groups (20, 40 mg/kg, i.g.) and the DEX groups were decreased significantly.

### 2.3. SAA Improved 5/6 Nephrectomized Model-Induced Chronic Kidney Disease

**Figure 2:**
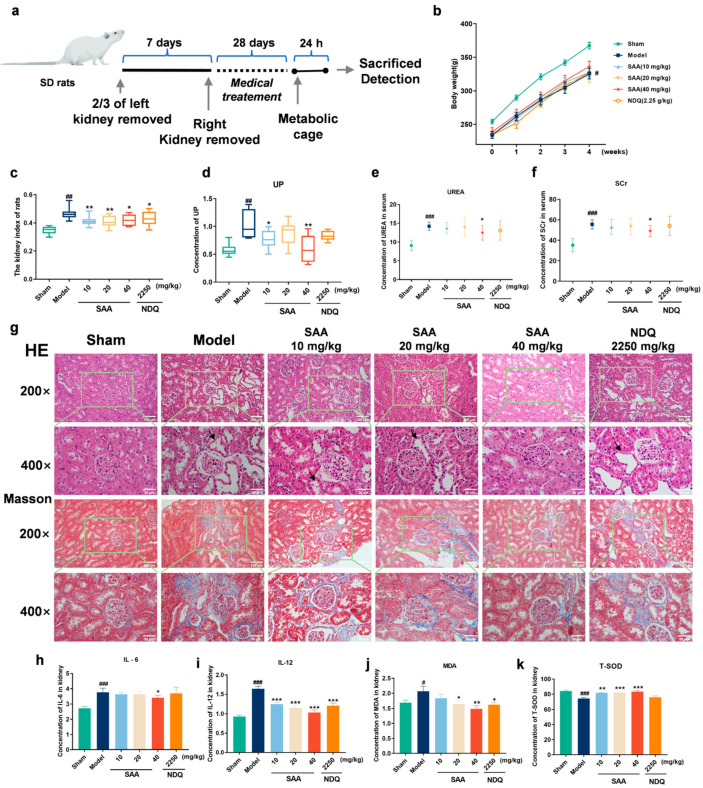
The effects of SAA on CKD rats. (**a**) SAA suppressed kidney injury in CKD rats. (**b**–**d**) The effects of SAA on (**b**) the body weight, (**c**) the kidney index (*n* = 10–12, # *p* < 0.05, ## *p* < 0.01 compared with Sham group. * *p* < 0.05, ** *p* < 0.01 compared with Model group), and (**d**) the content of UP. *n* = 8–14. (**e**,**f**) Effect of SAA on serum (**e**) UREA and (**f**) SCr in rats with CKD (*n* = 4–6, ## *p* < 0.01, ### *p* < 0.001 compared with Sham group. * *p* < 0.05, ** *p* < 0.01 compared with Model group). (**g**) Kidney histopathological changes and tubulointerstitial fibrosis were examined by H&E staining and Masson staining in kidney sections of CKD rats (*n* = 3, magnification: 200×, 400×, scale bar = 50 μm), black arrow indicates kidney tubular dilatations. All data are shown as mean ± SEM, *n* = 8–14. # *p* < 0.05, ## *p* < 0.01, ### *p* < 0.001 compared with Sham group. * *p* < 0.05, ** *p* < 0.01 compared with Model group. (**h**–**k**) Effect of SAA on the content of (**h**) IL-6, (**i**) IL-12, (**j**) MDA and (**k**) T-SOD in the kidneys of CKD rats. All data are shown as mean ± SEM, *n* = 4–6. # *p* < 0.05, ### *p* < 0.001 compared with Sham group. * *p* < 0.05, ** *p* < 0.01, *** *p* < 0.001 compared with Model group.

To further explore the efficacy of SAA, the 5/6Nx model was used to establish CKD rats [16], and the experimental design is shown in Figure 2a. The changes in body weight and the kidney index in CKD rats are shown in Figure 2b,c. After four weeks of administration, SAA had no significant effect on the body weight. Compared with the Control group, the kidney index of the rats in the Model group was significantly increased. Compared with the Model group, the SAA groups and the NDQ (Niaoduqing granules) group significantly decreased kidney index.

The content of UP in 5/6Nx rats is shown in Figure 2d. Compared with the Control group, the UP content of the Model group rats increased significantly, and compared with the Model group, the UP content of the SAA (10, 40 mg/kg, i.g.) group rats was significantly decreased. The levels of UREA and SCr in the serum of 5/6Nx rats in each group are shown in Figure 2e,f. Compared with the Control group, the UREA and SCr contents of the Model group increased significantly, and compared with the Model group, the UREA and SCr contents of the SAA groups (40 mg/kg, i.g.) were significantly decreased.

As shown in Figure 2g, the effect of SAA on kidney injury in 5/6 Nx rats was investigated by histological examination of kidney slices. HE staining showed that the physiological structure of the kidney in the Sham group was normal; the Model group showed severe tubulointerstitial lesions, including tubular atrophy, dilation and increased tubular basement membrane thickness. In the SAA groups, kidney injury was significantly improved, and the cell arrangement and tubular structure of the kidney returned to normal. Masson staining was used to detect tubulointerstitial fibrosis. The results showed that the Control group had no tubulointerstitial fibrosis; the rats in the Model group had obvious tubulointerstitial fibrosis, and the degrees of kidney tubulointerstitial fibrosis in the SAA groups reduced significantly.

### 2.4. SAA Inhibited the Release of Inflammatory Cytokines and Anti-Oxidative Stress in 5/6Nx Rats

The contents of IL-6, IL-12, MDA and T-SOD in the kidneys of each group are shown in Figure 2h–k. Compared with the Control group after four weeks of administration, IL-6 and IL-12 levels in the Model group were significantly increased. Compared with the Model group, the level of IL-6 in the SAA groups (40 mg/kg, i.g.) was significantly decreased, and the level of IL-12 in the SAA (10, 20, 40 mg/kg, i.g.) and NDQ groups decreased significantly. Compared with the Control group, MDA was significantly increased and T-SOD was significantly decreased in the Model group. Compared with the Model group, the MDA in the SAA (20, 40 mg/kg, i.g.) and NDQ groups decreased significantly, and the T-SOD in the SAA group was significantly increased and statistically unchanged in the NDQ group. The results suggest that SAA can improve kidney injury by inhibiting the release of inflammatory cytokines and anti-oxidative stress in 5/6Nx rats.

### 2.5. Network Pharmacology Suggested That MAPKs and TGF-β1/Smads Signaling Pathways Were Key Mechanisms of SAA in the Treatment of CKD

SAA targets were analyzed in SwissTarget Prediction database (http://www.swisstargetprediction.ch/, accessed on 31 March 2022), GeneCards database (https://www.genecards.org/, accessed on 31 March 2022) and TCMSP database (https://tcmspw.com/tcmsp.php/, accessed on 31 March 2022) [29,30,31], the results were pooled and 237 target genes were obtained as shown in Supplementary Materials Table S1.

Disease-related targets were searched through the Online Mendelian Inheritance in Man (OMIM) database (https://www.omim.org/ accessed on 31 March 2022), DisGeNET database (https://www.disgenet.org/web/DisGeNET/menu/home/, accessed on 31 March 2022) and GeneCards database [32,33]. In the analysis with “chronic kidney disease” (CKD) as the keyword, 1361 targets were obtained as shown in Supplementary Materials Table S2.

**Figure 3:**
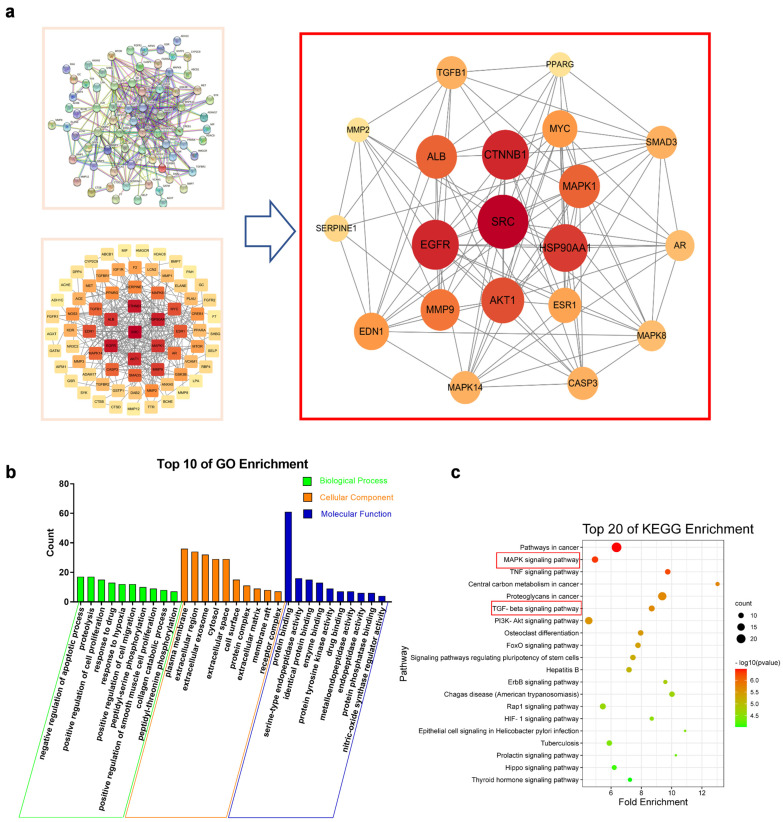
The pathway of SAA in treating 5/6Nx-induced CKD. (**a**) PPI plot of the relationship between CKD targets and SAA. (**b**) GO enrichment including the biological process (green), cellular component (red) and molecular function (blue). (**c**) KEGG pathway analysis of differential genes in Model group and SAA group.

Based on the above results, as shown in Supplementary Materials Table S3 the SAA core active targets were matched with the disease targets of CKD to obtain the compound targets of SAA-CKD. The above targets were imported into the String online (https://string-db.org/, accessed on 31 March 2022) with 0.7 confidence level to obtain the PPI network [34], then the medians of Betweenness Central-ity, Closeness Centrality and Degree Centrality of PPI were analyzed through the network topology analysis function of Cytoscape3.6.1 (https://www.cytoscape.org/, accessed on 31 March 2022) to obtain the core targets. According to the degree value, the top 20 core targets were screened, as shown in Figure 3a. The results of the GO enrichment analysis of differential genes are shown in Figure 3b. Regarding Biological Process, Cellular Component and Molecular Function, the top 10 items were screened out according to the *p*-value. Among them, the Cellular Com-ponent results such as cell membrane and extracellular body may be related to the infor-mation transmission pathway, which together with the Biological Process and Molecular Function such as enzyme binding and protein phosphorylation suggested that SAA may affect protein binding or phosphorylation by influencing the binding of extracellular lig-ands to cell membrane receptors, thus acting as a therapeutic agent for CKD.

KEGG pathway enrichment analysis of 80 intersecting genes was performed using the DAVID 6.8 database (https://david.ncifcrf.gov/, accessed on 31 March 2022). The bubble map of the first 20 KEGG metabolic pathways is shown in Figure 3c, suggesting that the MAPKs signaling pathway and TGF-β1/smads signaling pathway may be related to the SAA in the treatment of CKD.

### 2.6. SAA Affected TGF-β1/smads Protein Expression in the Kidneys of CKD Rats

**Figure 4:**
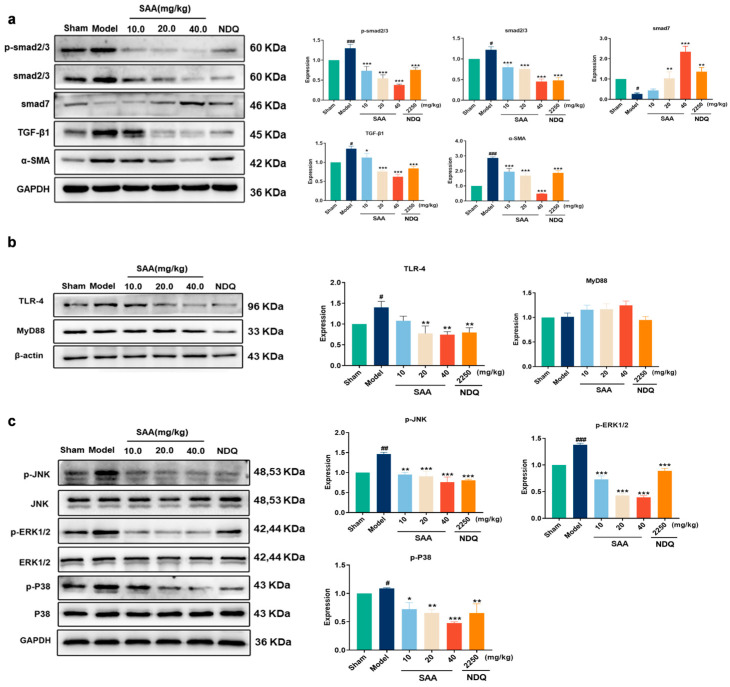
The effects of SAA on the expressions of TGF-β1/smads, TLR4/MyD88 and MAPKs signaling pathways in CKD rats. (**a**) Changes in TGF-β1/smad2/3 signaling pathway. (**b**) Changes in TLR4/MyD88 signaling pathway. (**c**) Changes in MAPKs signaling pathway. All data are shown as mean ± SEM, *n* = 3. # *p* < 0.05, ## *p* < 0.01, ### *p* < 0.001 compared with Sham group; * *p* < 0.05, ** *p* < 0.01, *** *p* < 0.001 compared with Model group.

The results are shown in Figure 4a. Compared with the Sham group, the α-SMA, TGF-β1, smad2/3, and p-smad2/3 expressions in kidneys of Model group rats were significantly increased, and the smad7 expression was significantly decreased. After four weeks of SAA administration, compared with the Model group, the expressions of α-SMA, TGF-β1, smad2/3, and p-smad2/3 were significantly decreased, and the expression of smad7 was significantly increased in the kidney tissues of the SAA groups (20, 40 mg/kg, i.g.) and NDQ group.

### 2.7. SAA Inhibited TLR4 Expression in the Kidneys of CKD Rats

The results are shown in Figure 4b. Compared with the Sham group, the expression of TLR4 in the kidneys of 5/6Nx rats was significantly increased in the Model group. Compared with the Model group, the expression of TLR4 was significantly decreased in the SAA groups (20, 40 mg/kg, i.g.) and NDQ group. SAA did not affect MyD88 expression in the kidneys of 5/6Nx rats.

### 2.8. SAA Inhibited the Activation of the MAPKs Signaling Pathway

The results are shown in Figure 4c. The expressions of ERK1/2, p38 and JNK did not change in the Control and Model groups. Compared with the Sham group, the expressions of p-ERK1/2, p-p38 and p-JNK in the Model group rats were significantly increased, and compared with the Model group, the expressions of p-ERK1/2, p-p38 and p-JNK in the SAA groups and NDQ group were significantly reduced. The results showed that the JNK, ERK1/2 and p38 kinases of the MAPKs signaling pathway in the kidneys of 5/6Nx rats were activated by inflammatory stimulation, and SAA significantly inhibited the phosphorylation of related proteins in the kidneys of 5/6Nx rats.

## 3. Discussion

SCr, UP and UREA levels and urine analysis are commonly used as biomarkers to assess kidney function [35,36,37,38]. Current studies have shown that the SCr level does not identify AKI and may be influenced by non-kidney diseases [39]. KIM-1 and NGAL are novel biomarkers of kidney injury and are promising as a way to predict AKI before Scr elevation [40,41]. NGAL is a secreted polypeptide that is protease resistant and therefore easily detected in urine. Studies have shown that NGAL in the kidney and urine is significantly up-regulated within 24 h after kidney ischemia [12]. NGAL levels have also been shown to be highly predictive of AKI in emergency room patients and cardiac surgery patients [42,43]. Studies have shown that KIM-1 mRNA and protein levels are significantly increased in the kidney after ischemia [11,44,45]. In the toxicity models of cisplatin, gentamicin and cyclosporine, KIM-1 significantly outperformed Scr and BUN as an early marker [46]. In this study, SAA significantly reduced UP, KIM-1 and NGAL levels in AKI rats, as well as significantly reduced UP, UREA and Scr levels in CKD rats, thus alleviating kidney injury.

Oxidative stress and inflammation play a key role in the development of CKD and are two major pathological mechanisms of CKD [47]. IL-6 and IL-12 play an important role in the inflammatory response as pro-inflammatory factors [48,49]. The degree of oxidative stress is positively correlated with the level of MDA, and the increase in T-SOD is to inhibit the damage caused by oxidative stress [50]. In this study, SAA significantly reduced the levels of IL-6, IL-12 and MDA and significantly increase the level of T-SOD, suggesting that SAA has significant anti-oxidative stress and anti-inflammatory activities. Meanwhile, SAA significantly improved the histopathological injury of the kidney in AKI rats and histopathological injury and the degree of kidney tubulointerstitial fibrosis in CKD rats by HE and Masson staining.

TGF-β was a cytokine with multiple pathological and physiological functions. The main isomers of the TGF-β family, TGF-β1, TGF-β2 and TGF-β3, show different biological activities [51,52]. Studies have shown that TGF-β1 is an indispensable immunoregulator promoting CKD progression by controlling the activation, proliferation and apoptosis of immunocytes [53]. The Smad2 and Smad3, primary downstream mediators of TGF-β1, are extensively activated in fibrotic kidneys in CKD models [54]. Studies have shown that the TGF-β signaling pathway can be blocked by inhibiting smad2/3 phosphorylation, thus exerting its anti-fibrotic effect [55]. Activated myofibroblasts are one of the major stroma-secreting cell types and α-SMA is their characteristic protein, the amount of which is positively correlated with kidney fibrosis [56]. In our study, Western blotting showed that the expressions of α-SMA, TGF-β1, smad2/3 and p-smad2/3 in the SAA group were significant decreased compared with the Model group, suggesting that SAA plays an anti-kidney fibrosis role by inhibiting the TGF-β1/smads signaling pathway.

The TLR4/MyD88/MAPK pathway is one of the main cell membrane signal transduction pathways [57]. Studies have shown that TLR4 promotes kidney fibrosis through inflammasome activation in kidney epithelial cells and may also be involved in immune responses associated with CKD [58]. In kidney biopsies from people with CKD, TLR4 expression was found to be significantly associated with the pro-inflammatory marker MCP-1 and TGF-β1, suggesting that increased expression of TLR4 is an important feature of CKD [59]. In this study, SAA significantly decreased TLR4 expression in the kidneys of 5/6Nx rats but did not affect MyD88 expression, suggesting that the regulation of CKD rats by SAA may not be through the MyD88-dependent pathway.

TGF-β can rapidly activate JNK and p38 in a SMAD-independent manner via the activation of MAP kinase kinases (MKKs) [60,61]. Studies have shown that the MAPKs pathway regulates CKD as a non-smads signaling pathway and inhibits kidney fibrosis [62]. Inhibition of p38-MAPK significantly alleviated proteinuria, inflammation, glomerulosclerosis and interstitial fibrosis [63]. In addition, blocking ERK1/2 phosphorylation inhibits myofibroblast and macrophage expansion and also indirectly affects TGF-β1 secretion [64]. Recent studies have demonstrated an important requirement for JNK1 in promoting collagen deposition in models of fibrosis, and the lack of JNK1 suppresses the expression of pro-fibrotic genes [65]. It has been reported that JNK mediates inflammation in human kidney mesangial cells and that inhibition of JNK phosphorylation prevents the conversion of AKI to CKD [66]. Our results showed that JNK, ERK1/2 and p38 kinases of the MAPKs signaling pathway were activated by inflammatory stimulation in the kidneys of 5/6Nx model rats, and SAA significantly inhibited the phosphorylation of related proteins in the kidneys of 5/6Nx rats, suggesting that SAA improves CKD through the MAPKs signaling pathway.

## 4. Materials and Methods

### 4.1. Animals

Male SD rats weighing 240–260 g were obtained from the animal Center of Shenyang Pharmaceutical University (Shenyang, China; License: SCXK, Liaoning, 2020-0001). The animals were fed with commercial pellets, had access to water ad libitum and were allowed to adapt to the environment for one week prior to the experiments. The room temperature was set at 22 ± 2 °C and the room humidity was set at 50% ± 20%. The animals used in this research study were approved in accordance with the Animal Ethics Committee of Shenyang Pharmaceutical University and the regulations were consistent with the ethical requirements of laboratory animals in China.

### 4.2. AKI Model (Gentamicin-Induced Rats)

After one week of adaptive feeding, 72 rats were randomly divided into six groups, namely Control group (normal saline 10 mL/kg/d, i.p.), Model group (normal saline 10 mL/kg/d, i.g.), SAA groups (10, 20, 40 mg/kg/d, i.g.) and positive drug DEX group (dexamethasone, 0.1 mg/kg/d, i.g.). Intraperitoneal injection of gentamicin (100 mg/kg) was performed at 8:00 am., and drug administration was performed at 2:00 pm. for 7 consecutive days.

### 4.3. CKD Model (5/6 Nephrectomized Rats)

The 72 rats were acclimated for 1 week and then randomly divided into six groups as follows: group 1: the Sham group was subjected to sham operation; group 2: the 5/6Nx, in which the upper and lower poles of the left kidney were ablated, and subsequently, the right unilateral nephrectomy was performed 1 week later; groups 3, 4 and 5: SAA-treated 5/6Nx rats with SAA (10, 20, 40 mg/kg/d, i.g.); group 6: NDQ-treated 5/6Nx rats (2.25 g/kg/d, i.g.). All animals were operated on under anesthesia and given chloral hydrate solution (350 mg/kg, i.p.).

### 4.4. Kidney Index of Rats

The rats were killed after excessive anesthesia. The kidneys were rinsed with pre-cooled normal saline, dried with filter paper and weighed to calculate the kidney index.

*Kidney index* = *kidney weight*/*body weight* × 100%.

### 4.5. Histopathological Analysis

The isolated rat kidneys were fixed with 4% paraformaldehyde for more than 24 h and then dehydrated with a gradient with alcohol. After paraffin embedding the dehydrated tissue, the tissue was cut into 3 μm thick slices. Then, slices were dewaxed with xylene and stained with hematoxylin–eosin. After dehydration with ethanol, the slices were sealed. Finally, the samples were observed with an optical microscope with a magnification of 200 and 400.

### 4.6. Masson Staining

The paraffin slices were sequentially placed in gradient xylene solution and ethanol solution and then washed with water. We soaked the slices in potassium dichromate overnight and rinsed them under running water. After rinsing the samples with hematoxylin iron staining, the slices were placed in Ponceau red acid fuchsin for 5 min. After soaking in molybdate phosphate aqueous solution for 2 min, the slices were placed directly in aniline blue dye solution for 3 min. Slices were differentiated with 1% glacial acetic acid and dehydrated with absolute ethanol. Finally, slices were cleared with xylene and sealed with neutral gum.

### 4.7. Biochemical Analysis

The rats were fasted within 24 h after the last administration but could drink water freely. Blood was taken from the orbit and placed for 2 h at room temperature. Under the condition of 4 °C, the centrifuge was rotated at 3000 rpm/min and centrifuged for 15 min. The upper serum was absorbed and then sub-packed at −80 °C. Blood biochemical indicators (UREA and SCr) were detected by an automatic biochemical analyzer (HITACHI).

### 4.8. ELISA

The kidneys were weighed and the tissue was cut up and homogenized by adding PBS at a weight-to-volume ratio of 1:9. The homogenate was broken up by ultrasound. The homogenate was centrifuged at 2–8 °C, 1500× *g* for 10 min, and the supernatant was taken for MDA (Jiancheng, Nanjing, Jiangsu, China), T-SOD (Jiancheng, Nanjing, Jiangsu, China), IL-6 (Elabscience, Wuhan, Hubei, China) and IL-12 (Elabscience, Wuhan, Hubei, China) assay. According to the manufacturer’s instructions, KIM-1 and NGAL in serum were quantified using assay kits (Elabscience, Wuhan, Hubei, China). UP was quantified using assay kits (Jiancheng, Nanjing, Jiangsu, China).

### 4.9. Western Blotting

After the filter paper absorbed excess water, the kidney was placed in a 2 mL EP tube, and 100 μL of RIPA (PMSF: NaF: Na_3_VO_4_: RIPA = 1:1:1:100) was added to each 10 mg of tissue. We shredded the tissue and fully lysed it with a sonicator, set the intensity to 20% and the time to 3 min. This was repeated twice. After standing on ice for 30 min, it was centrifuged at 12,000 rpm for 10 min at 4 °C. Using the BCA method to determine the protein concentration, followed by SDS-polyacrylamide gel electrophoresis (SDS-PAGE), separate gels of different concentrations and 5% concentrated gels were formulated according to different proteins.

### 4.10. Network Pharmacology

SwissTarget Prediction (http://www.swisstargetprediction.ch/, accessed on 31 March 2022), GeneCards (https://www.genecards.org/, accessed on 31 March 2022), TCMSP databases (https://tcmspw.com/tcmsp.php/, accessed on 31 March 2022), Online Mendelian Inheritance in Man (OMIM) (https://www.omim.org/, accessed on 31 March 2022) and DisGeNET database (https://www.disgenet.org/web/DisGeNET/menu/home/, accessed on 31 March 2022) were searched to identify all the target genes of SAA and CKD to construct the PPI network. GO and KEGG pathway enrichment analyses were performed using DAVID 6.8.

### 4.11. Statistical Analysis

All results were presented as mean ± SEM, both one-way ANOVA and Tukey’s test were used for comparisons between groups, and *p* < 0.05 was taken as the standard of significant difference. All statistical analyses were performed using SPSS 22.0 software and Graphpad Prism 8.02.

## 5. Conclusions

In this study, SAA improved gentamicin-induced AKI and 5/6 Nx-induced CKD, which may be achieved by inhibiting the release of inflammatory factors and alleviating oxidative stress injury, as well as regulating the MAPKs and TGF-β1/smads signaling pathways.

## Data Availability

Data is contained within the article or Supplementary Material.

## Supplementary Materials

The following supporting information can be downloaded at: https://www.mdpi.com/article/10.3390/molecules28083630/s1, Table S1: The target genes of SAA; Table S2: The target genes of CKD; Table S3: target genes of CKD and SAA.

## Abbreviations

SAA	Salvianolic acid A
AKI	Acute kidney injury
CKD	Chronic kidney disease
5/6Nx	5/6 nephrectomized
KIM-1	Kidney injury molecule-1
NGAL	Neutrophil gelatinase-associated lipocalin
SCr	Serum creatinine
UREA	Urea
UP	Urine protein
IL-6	Interleukin 6
IL-12	Interleukin 12
MDA	malondialdehyde
T-SOD	Total superoxide dismutase
ERK	Extracellular regulated protein kinases
MAPKs	Mitogen-activated protein kinase signaling
TGF-β	Transforming growth factor-beta
PKD	Polycystic kidney disease
LPS	Lipopolysaccharide
STZ	Streptozocin
DEX	Dexamethasone
α-SMA	α-Smooth Muscle Actin
NDQ	Niaoduqing granules
MKKs	MAP kinase kinases
