# Supplementary material for: Salvianolic Acid A Improves Rat Kidney Injury by Regulating MAPKs and TGF-β1/Smads Signaling Pathways
Source: Molecules. 2023 Apr 21;28(8):3630. doi: 10.3390/molecules28083630 (PMC10144349; doi:10.3390/molecules28083630)
Supplement: Supplementary file 1 [file molecules-28-03630-s001.zip › molecules-2308592-supplementary.pdf]

Table 1. The target genes of SAA.

TCMSP	Swiss Target Prediction	Genecards		237 SAA target genes						
F10	AKR1B10	PPARA	MET	MAPK14	GSTP1	MAOB	ISG20	HSPA8	ADH1B	ACP3
F7	FYN	CASP3	ATF2	CFB	EGFR	PPARG	AMD1	ADAM17	LDHB	ESRRG
PTPN1	AKR1B1	MYC	EIF2AK3	MAPKAPK2	TREM1	PDE4B	PTPN1	APCS	B3GAT1	PDHB
PRSS1	TDP1	MAPK1	ACHE	FAP	RIDA	CDK2	FABP4	HSD11B1	TYMS	PCK1
AKT1	AKR1B15	NOS3	BMP7	BCHE	DDX6	AKR1B1	MMP8	SORD	MMP13	HDAC8
CCND1	AKR1A1	MMP2	PHB1	STS	RTN4R	CTSS	PAH	MTHFD1	LPA	AKR1C1
BCL2L1	AKR1E2	HMGCR	SMAD3	APOA2	MMP3	BACE1	DHODH	AR	NR3C2	SULT2A1
CDKN1B	YES1	ABCB1	PRMT1	CDK5R1	ESR2	CA12	F10	GATM	PARP1	ADK
EIF6	FGR	MTOR	VLDLR	CMA1	PRDX5	QPCT	IGLV2-8	PPARD	ARF1	PDE5A
CASP3	SRC	NR1I2	SIK2	TTR	GBA	CHEK1	CHIT1	YARS1	CBR1	
	FRK	AKT1	DAB2	CA2	PPIA	LCN2	PRKACA	PNPO	C1R	
	MMP1	MAPK14	TEP1	PNP	SELP	FCAR	PDPK1	KIF5B	CRAT	
	MMP2	HSPA5	ERP29	ESR1	ANG	AKR1C3	PLK1	AZGP1	CDK6	
	MMP3	VCAM1		NPR3	ICAM2	DAPK1	ADH1C	TYMP	FGFR2	
	MMP9	MAPK3		MAPK10	MIF	GSR	PPP5C	HSD17B1	NMNAT3	
		MAPK8		MAPK8	CASP3	KIF11	EEA1	GC	GSK3B	
		TGFB1		ALB	NQO2	CTSB	ME2	LGALS7	DHFR	
		MMP1		NR1H2	CA1	AMY1A	BCAT2	LGALS7B	AHCY	
		ACE		PIM1	NOS3	AMY1B	SHBG	ADH5	SYK	
		CTNNB1		SRC	TGFBR2	AMY1C	BMP7	RXRA	FNTA	
		ATF6		CLPP	HSD17B11	CASP7	KDR	NQO1	IMPDH2	
		MIR106B		PGR	MAPK1	PDE4D	CSNK2A1	RAC2	PLAU	
		CDH5		CCNA2	CTSD	MTAP	LTF	SEC14L2	DPP4	
		TAGLN2		SNRPA	CSNK1G2	FGFR1	CFD	MMP12	LTA4H	
		DUSP6		F2	CTSV	TGFBR1	ANXA5	PYGL	CDA	
		MIR660		EPHB4	HSP90AA1	KYAT1	IGF1R	PSPH	LCK	
		SERPINE1		FRK	MAPK3	AIFM1	CDH5	CCND1	AKR1C2	
		SHC1		MMP1	TGFB1	SIRT1	TAGLN2	BCL2L1	ELANE	
		GOT1		MMP2	ACE	ATF2	DUSP6	CDKN1B	MET	
		ATF4		MMP9	CTNNB1	EIF2AK3	DAB2	EIF6	CTSK	
		PPIA		PPARA	SERPINE1	ACHE	TEP1	AKR1B10	RBP4	
		CREB1		MYC	SHC1	PHB1	ERP29	FYN	CYP2C9	
		EIF4EBP1		HMGCR	GOT1	SMAD3	MIR660	TDP1	DCK	
		EDN1		ABCB1	ATF4	PRMT1	OTC	AKR1B15	UCK2	
		AKR1B1		MTOR	CREB1	VLDLR	F7	AKR1A1	AGXT	
		RB1		NR1I2	EIF4EBP1	SIK2	PRSS1	AKR1E2	PLA2G2A	
		AIFM1		HSPA5	EDN1	ATF6	AKT1	YES1	CTNNA1	
		SIRT1		VCAM1	RB1	MIR106B	AKT1	FGR	ARSA	

Table 2. The target genes of CKD.

**1361 CKD target genes**						
PKD1	*SERPINA1*	*BRCA2*	*SLC17A5*	*CRB2*	*MIR145*	*FGFR3*
PKHD1	*ABL1*	*DYNC2H1*	*LOC106627981*	*CPT2*	*ENPP1*	*HLA-DQA1*
PKD2	*NPHP3*	*STAT1*	*G6PC1*	*PRKCSH*	*IFNA1*	*PSEN2*
TP53	*ALG9*	*NPHS2*	*B2M*	*IL2RA*	*ABCB1*	*EGF*
TNF	*ATM*	*NAGLU*	*PTEN*	*MTHFR*	*GBE1*	*IL1RN*
IL6	*CRP*	*EGFR*	*RYR1*	*IL2*	*IL18*	*CCR6*
CYBB	*MMP1*	*CCND1*	*FAS*	*MIR1225*	*TNFRSF1A*	*APOB*
UMOD	*INS*	*APOL1*	*BCR*	*PARK7*	*FIG4*	*NPPA*
HNF1B	*GLA*	*KRAS*	*SOD1*	*PIK3CA*	*CST3*	*C3*
IL10	*SNCA*	*HLA-DQB1*	*VCP*	*FLNA*	*NOTCH1*	*ANO5*
TGFB1	*PAX2*	*MIR21*	*PMP22*	*MTOR*	*GARS1*	*HRAS*
ACE	*IL1B*	*DZIP1L*	*AKT1*	*IFT172*	*JAG1*	*SOX10*
APOE	*NPC1*	*LRP5*	*NEFL*	*COL4A1*	*HLA-A*	*BCL2*
TSC2	*NCF4*	*PTPN22*	*NPHP4*	*FCGR2A*	*CUBN*	*NPC2*
CYBA	*NOS3*	*SQSTM1*	*GDNF*	*MIR155*	*MIR106B*	*THBD*
GANAB	*CFH*	*TMEM67*	*CXCL8*	*HLA-DPB1*	*ERBB2*	*MIR20A*
NCF2	*MPZ*	*VHL*	*MEFV*	*FGF23*	*EDN1*	*MIR6511B1*
CEP290	*MKS1*	*INF2*	*PDGFRB*	*SEC63*	*OFD1*	*AQP2*
CTLA4	*NLRP3*	*PRKN*	*FBN1*	*IQCB1*	*MAPK1*	*COMT*
HLA-DRB1	*STAT3*	*TERT*	*CTNNB1*	*HEXA*	*SLC34A1*	*NRAS*
RET	*DNAJB11*	*APOA1*	*IFT140*	*SETBP1*	*RUNX1*	*APC*
COL4A5	*NPHP1*	*MAPT*	*GDAP1*	*MME*	*IGHMBP2*	*FOXP3*
NOD2	*TSC1*	*CCL2*	*AGT*	*PON1*	*MIR143*	*MET*
GBA	*CLCN5*	*TTC21B*	*KIT*	*ABCA1*	*HSPB1*	*NF1*
CFTR	*PRNP*	*MPO*	*GJB1*	*BICC1*	*MIR126*	*NPPB*
MUC1	*MIR15A*	*TTR*	*HBB*	*DSTYK*	*IGF1*	*EDNRA*
REN	*TRPV4*	*JAK2*	*HMOX1*	*PPARG*	*SLC37A4*	*GNAS*
COL4A4	*HFE*	*INVS*	*EPO*	*PSAP*	*HNF1A*	*MYH9*
GATA3	*SMPD1*	*AGTR1*	*MMP9*	*ICAM1*	*SPP1*	*LEP*
NCF1	*ATP7B*	*FN1*	*IL4*	*DYNC1H1*	*INPP5E*	*KIF1B*
WT1	*VEGFA*	*ABCA4*	*COPA*	*ALMS1*	*CCR5*	*PTPN11*
GAA	*BRAF*	*SH3TC2*	*EDNRB*	*TLR2*	*ELN*	*SBF2*
ALB	*VWF*	*POLG*	*F2*	*MIR29A*	*MIR146A*	*GPT*
IFNG	*MFN2*	*NPHS1*	*TNFRSF11B*	*AGL*	*CFHR5*	*MT-ND1*
MIR17	*TLR4*	*PRX*	*WDR19*	*MIR223*	*IL13*	*LCN2*
PSEN1	*LRRK2*	*ACTN4*	*MIR34A*	*AHI1*	*HTT*	*LOC106029312*
CC2D2A	*COL4A3*	*PINK1*	*EDN3*	*BDNF*	*IL17A*	*CCL5*
APP	*HLA-B*	*PRTN3*	*IRF5*	*NCF4-AS1*	*DSP*	*ADIPOQ*
LMNA	*CASR*	*BSND*	*CLCNKB*	*VDR*	*NDRG1*	*AARS1*
MMACHC	*SMAD4*	*CFI*	*TET2*	*PYGM*	*MT-ATP6*	*MYC*

CD79A	*F5*	*OCRL*	*LCAT*	*PTGS2*	*HEXB*	*FGFR1*
LRSAM1	*LPL*	*SERPINA3*	*TNFRSF11A*	*TARDBP*	*CDH1*	*DNM2*
BBS1	*TLR5*	*SPINK1*	*CD40LG*	*RMND1*	*ADRB2*	*GFAP*
ESR1	*LIPA*	*LMX1B*	*SREBF1*	*MECP2*	*CHEK2*	*IGF2*
LDLR	*MTMR2*	*ABCB4*	*IL1A*	*GATA1*	*COL2A1*	*CP*
RBP4	*MT-TL1*	*CYBC1*	*MKKS*	*PDGFRA*	*ALPL*	*SEC61A1*
C4A	*LITAF*	*ASXL1*	*MMP2*	*G6PD*	*BSCL2*	*FLT1*
MBL2	*SMN1*	*CD4*	*SDHB*	*MT-CYB*	*TBX18*	*NOTCH2*
NEK1	*ADAMTS13*	*BRCA1*	*NEK8*	*ADAM17*	*SLC12A3*	*CDKN1C*
VCAM1	*RRM2B*	*FGFR2*	*IL10RA*	*PLP1*	*CD2AP*	*ATXN3*
MARS1	*CCL11*	*RPL36A-HNRNPH2*	*COL1A1*	*LRP2*	*ZAP70*	*CLEC7A*
FASLG	*TNFSF11*	*CASP3*	*KCNQ1*	*NFKB1*	*PRKAG2*	*HIF1A*
TF	*ACTA2*	*AIRE*	*PDCD1*	*ATXN2*	*CTNS*	*MUC5B*
ACP5	*HNRNPA1*	*AR*	*ENG*	*USH2A*	*ATP7A*	*RETN*
STAT4	*MIR107*	*SERPINE1*	*F8*	*UCHL1*	*CAT*	*CHAT*
FLCN	*PROM1*	*EGR2*	*IL23R*	*PMS2*	*HAVCR1*	*MEN1*
HLA-C	*MORC2*	*CDKN2A*	*SLC11A1*	*NOS2*	*LAMP2*	*LOC107133510*
PKD2L1	*HNF4A*	*CFHR1*	*RPGRIP1L*	*CXCR4*	*CASP8*	*NTRK1*
HP	*TRAF3IP1*	*ADA*	*MIF*	*AVPR2*	*MMP3*	*HAMP*
ELANE	*IL5*	*H19*	*KL*	*CXCL12*	*ATP2A2*	*CAV1*
CD40	*TREX1*	*NPHP3-ACAD11*	*CCN2*	*PLAU*	*WFS1*	*MFF-DT*
TNFRSF1B	*MIR203A*	*CD36*	*WDR35*	*SDCCAG8*	*CLN3*	*P2RX7*
BMP4	*SLC4A1*	*DGKE*	*PKD1L1*	*ECE1*	*STAT5B*	*SETD2*
KCNJ11	*AGXT*	*CXCL10*	*INSR*	*JUP*	*PKD3*	*PTH*
MPL	*GLIS2*	*MT-TK*	*DMD*	*SYNJ1*	*TTN*	*CSF3*
SLC22A12	*SMAD3*	*GHRL*	*APRT*	*MIR122*	*MED25*	*TGFBR2*
PRKAR1A	*GALC*	*MIR29C*	*PKD2L2*	*NSD1*	*MT-CO1*	*SLC2A9*
GPC3	*CSF2*	*SAG*	*JUN*	*CLLS1*	*TMEM231*	*ITGAM*
CSF3R	*IL17F*	*FCGR3B*	*GLB1*	*CLLS3*	*TFRC*	*FGD4*
SST	*ABCC8*	*PIK3R1*	*GP1BA*	*FRAS1*	*ADA2*	*IFT80*
FGF2	*IDUA*	*SURF1*	*TIMP1*	*FLG*	*ALG8*	*SLC9A3*
PBX1	*NR3C1*	*SAA1*	*PTPRC*	*DBT*	*TGFB2*	*SFTPC*
BCKDHB	*ARSA*	*SNCAIP*	*CYP27A1*	*ITGA8*	*TG*	*AGER*
MYH7	*SH2B3*	*IRF8*	*F9*	*GRN*	*COL7A1*	*TMEM216*
OPTN	*SELP*	*CALCA*	*ABCB11*	*BAX*	*SLC2A2*	*TRPC6*
IFNA2	*CLCNKA*	*BCS1L*	*KCNN4*	*SLC12A1*	*POMC*	*PHKA2*
CEP164	*PLG*	*CLLS4*	*PYGL*	*GYS1*	*ABCD1*	*BMPR2*
TTC21B-AS1	*DES*	*CLLS5*	*PAX6*	*SCN5A*	*SLC26A1*	*BCKDHA*
LOC106099062	*ATP2C1*	*EP300*	*SDHD*	*EYA1*	*NR3C2*	*HLA-G*
SHH	*HMGB1*	*TH*	*NDUFAF6*	*IFNGR1*	*PLEKHG5*	*LTA*
DCDC2	*NOTCH3*	*RAF1*	*HBA2*	*ABCA3*	*CD46*	*F3*
GATA4	*H2AC18*	*PRF1*	*LOC107982234*	*KAT6B*	*SMARCAL1*	*BGLAP*
IFIH1	*HPRT1*	*CDKN1A*	*ACTA1*	*BBS10*	*RIPK1*	*GJB2*

C9orf72	*KMT2A*	*GCK*	*LIPC*	*CALR*	*IL7R*	*PRPS1*
GGT1	*MT-ND5*	*NDP*	*ERCC6*	*MITF*	*SPTLC1*	*MIR10A*
CREBBP	*PRKCD*	*BBS4*	*SCARB2*	*PEX6*	*MT-ND6*	*MYH11*
MIR132	*MT-ND4*	*THPO*	*MIR210*	*PRSS1*	*FUS*	*APBB1*
ASPA	*CTSD*	*MIR181A2*	*TBK1*	*PLA2G7*	*ALOX5*	*ETV6*
LPA	*LDHA*	*ERBB3*	*TYMP*	*SOST*	*MIR140*	*TWNK*
PFKM	*TP63*	*MT-ND2*	*TBX5*	*COQ8B*	*FAM13A*	*NLRP1*
TBX1	*GSTM1*	*FGF8*	*CYP27B1*	*COL1A2*	*STX1A*	*NR4A2*
MIR141	*GSTP1*	*RELA*	*RPGR*	*BTK*	*XIAP*	*SLPI*
BBS2	*TREM2*	*ITGB3*	*BMP7*	*MIR424*	*CACNA1A*	*U2AF1*
GPC4	*SLC19A3*	*REST*	*GM2A*	*ACTG2*	*NRIP1*	*KRT7*
ANKS6	*CCL3*	*MIR125A*	*SERPINC1*	*WNT4*	*SCO2*	*LOC105371046*
ATRX	*HSPB8*	*MIR199A1*	*PIK3CD*	*KAAG1*	*YARS1*	*LOC110006319*
NGF	*CD28*	*MIR221*	*FCGR2B*	*PIK3C2A*	*IL17RA*	*CYP2D6*
ZEB2	*PTCH1*	*IL1R1*	*MYD88*	*SOX9*	*SOD2*	*FAN1*
CLCN1	*SELE*	*SFTPA1*	*SMARCA4*	*HBA1*	*ABCG5*	*JAK1*
MYLK	*RYR2*	*ICOSLG*	*BMP6*	*KRT18*	*CDH23*	*KITLG*
CD8A	*RUNX2*	*CARD9*	*SLC2A1*	*POMGNT1*	*GAPDH*	*TLR9*
PKD1-AS1	*ACE2*	*FLT3*	*IL2RB*	*AVP*	*NKX2-5*	*CLCN7*
KIAA0319L	*TSHR*	*IL12RB1*	*AQP1*	*FANCD2*	*ZNF423*	*SLC6A4*
MIR192	*GDF6*	*HNFJ3*	*PRPH2*	*IL12A*	*SCNN1A*	*FGA*
PKDREJ	*IGF1R*	*TNFSF13B*	*MIR16-1*	*RPGRIP1*	*CX3CR1*	*TNNT2*
LAMB2	*DNAJB2*	*SYK*	*CLDN16*	*KDR*	*FREM2*	*OGG1*
CETP	*VIM*	*HSPD1*	*SMARCB1*	*JAK3*	*TNFAIP3*	*TNNI3*
PCSK9	*NUP107*	*C4B*	*CHRNA3*	*MT-CO3*	*NFE2L2*	*AHSG*
IL12B	*ADM*	*CD274*	*GNB3*	*BEST1*	*POT1*	*MYCN*
IGHG1	*F12*	*ABCC2*	*SRSF2*	*SLC6A3*	*MIR142*	*CNGB3*
PDGFB	*EHHADH*	*SLC25A4*	*CCR1*	*BMP2*	*GIGYF2*	*SLC22A5*
CD19	*CD14*	*IGFBP3*	*PKD1L2*	*SP110*	*CYP1B1*	*MSH2*
HSPA4	*AIFM1*	*SMN2*	*MVK*	*MBTPS2*	*HSPG2*	*LEPR*
NAGA	*ADAR*	*KNG1*	*TBP*	*RAG1*	*FHIT*	*PPARGC1A*
MGP	*ITCH*	*CXCR3*	*BBS12*	*GSN*	*PALB2*	*SFTPB*
COL3A1	*IRF4*	*PRMT7*	*LBR*	*FCGR3A*	*CDKN2B*	*MIR29B1*
CD34	*PHOX2B*	*PRKCQ*	*BLK*	*ACTC1*	*CBL*	*NFKBIA*
RNLS	*RNF213*	*DNAH5*	*SMAD6*	*ADH1C*	*PDX1*	*TERC*
GLE1	*MAP2K1*	*LIG4*	*HLA-DPA1*	*PSMB8*	*PRL*	*MIR19A*
HNRNPA2B1	*HFE-AS1*	*MT-ND3*	*COL17A1*	*EPM2A*	*PAX8*	*CFHR2*
ADD1	*HSP90AA1*	*B9D1*	*DCTN1*	*IL3*	*DNAJC13*	*MMUT*
BBS5	*GATM*	*RNASE3*	*IL15*	*SELL*	*NBN*	*MLH1*
GRHPR	*XPNPEP3*	*GLI3*	*LIFR*	*VPS35*	*PTPRO*	*CD81*
DNASE1	*EPCAM*	*FAT4*	*ITGB2*	*CAV3*	*CFHR3*	*SLC5A2*
EVC2	*PSTPIP1*	*SCT*	*MIR150*	*CIITA*	*MIR144*	*ABCC6*
HTRA2	*XDH*	*ACTB*	*IL10RB*	*POLR2F*	*SGPL1*	*PEX1*
CFTR-AS1	*MAPK14*	*PKD1L3*	*MPV17*	*IL7*	*TMEM237*	*AFP*

DNMT1	*ROBO2*	*MIR27A*	*TYR*	*ATG16L1*	*TLR3*	*HNRNPUL2-BSCL2*
SP140	*HTR2A*	*IFT43*	*BCL2L1*	*CD38*	*GNB1*	*CSPP1*
KIAA0586	*IL6R*	*CEACAM3*	*CR1*	*YWHAE*	*VPS13C*	*SPG11*
MSH6	*RHO*	*KARS1*	*HSD11B2*	*PON2*	*CYCS*	*FKRP*
POLR1C	*ERCC2*	*PLAT*	*SCN4A*	*XK*	*SLC7A7*	*GUSB*
ARHGDIA	*MEG3*	*CASP9*	*BCHE*	*DPP4*	*MAGI2*	*PLCE1*
TNFSF10	*FABP1*	*ABCA7*	*TCTN3*	*FH*	*IL21*	*FLT4*
RAD51C	*LOC111674472*	*IRAK1*	*ERAP1*	*MYH6*	*SCNN1G*	*SGSH*
ATRIP	*IKZF3*	*LGALS3*	*ANXA5*	*SAMHD1*	*FANCI*	*RAG2*
DLD	*PHEX*	*ERBB4*	*CYP3A5*	*ATP8B1*	*TCTN2*	*GLIS3*
CD80	*BBS9*	*IGFBP1*	*CLU*	*TRAF3IP2*	*RPS15*	*GATA2*
CASP1	*DNMT3A*	*GUCY2D*	*SH2D1A*	*KCNJ10*	*ATP6V1B1*	*TRIP13*
SDHC	*OPA1*	*CYP11B2*	*CD247*	*ASAH1*	*KRT8*	*NDUFS4*
CLLU1	*HARS1*	*PEX5*	*MIR200C*	*IL4R*	*CEP120*	*PEX7*
SFTPD	*SCNN1B*	*CCND2*	*CHGA*	*EMP2*	*ENO2*	*CTRC*
PPARA	*PAH*	*AGTR2*	*KRT5*	*SLC22A4*	*ABCG8*	*PEX12*
GJC2	*DRD2*	*DYRK1A*	*FIP1L1*	*HMGCR*	*CRB1*	*S100A9*
CDKN1B	*IL33*	*PODXL*	*C12orf29*	*IKBKG*	*ITGA3*	*GZMB*
HSD17B10	*ANLN*	*TGFB3*	*GCG*	*LTF*	*CDC73*	*PECAM1*
SOCS1	*FOS*	*MAN2B1*	*TPO*	*SDHA*	*SIX1*	*BBS7*
PNPLA3	*MYO1E*	*ELOVL4*	*STX3*	*MIR483*	*GJA1*	*PMM2*
CSF1	*STAT5A*	*DNAJC6*	*GLS*	*RNASEH2C*	*BCOR*	*B9D2*
MIR30A	*NCAM1*	*LOX*	*NEK9*	*DICER1*	*NR1H4*	*CRH*
RAD51	*ARID1B*	*NIPBL*	*IFT27*	*GC*	*IRS1*	*CELIAC5*
IFT122	*MLXIPL*	*ROBO1*	*MB*	*SPG7*	*UPK3A*	*SALL4*
CREB1	*FTL*	*MMP12*	*SLC3A1*	*RPL5*	*CFB*	*GSR*
HDAC8	*TRIM2*	*NEFH*	*CELIAC2*	*SOS1*	*PSENEN*	*RPE65*
MIR93	*FOXC1*	*S100A8*	*PTGS1*	*PEX2*	*RTEL1*	*ACHE*
BRIP1	*SYP*	*ADAM10*	*FGF10*	*TRIM28*	*SCN2A*	*LOC107303340*
APOC3	*KCNH2*	*TLR1*	*GCLC*	*CYP1A1*	*CD27*	*SFTPA2*
LOC107548112	*TCF4*	*IFNB1*	*SBF2-AS1*	*CD59*	*SLC26A4*	*F7*
COQ2	*NPY*	*PTH1R*	*COL11A1*	*APOH*	*FANCC*	*CELIAC6*
CTSB	*ACVRL1*	*NPM1*	*BTNL2*	*S100B*	*CELIAC10*	*CEP83*
PDE11A	*LZTFL1*	*SMAD2*	*TLR7*	*GRIN2B*	*CELIAC11*	*IKZF1*
NUP93	*KCNJ1*	*FREM1*	*NHLRC1*	*SLC6A19*	*CELIAC12*	*PRDM16*
GH1	*AMACR*	*MAPK8*	*GSK3B*	*SLC34A3*	*CELIAC7*	*CELIAC13*
CXCR2	*UGT1A1*	*SLC7A9*	*RBCK1*	*HNMT*	*CELIAC8*	*CLN6*
WAS	*SBF1*	*ATP13A2*	*MIR195*	*ALDOB*	*CELIAC9*	*KMT2D*
CYP2C9	*SMC1A*	*LRBA*	*ATP1A1*	*ALAD*	*PNPLA2*	*TTC8*
TNXB	*SLC9A3R1*	*CYP2E1*	*STXBP2*	*PHKB*	*CASP10*	*DYNC2LI1*
EIF4G1	*CDK12*	*CRYAA*	*NUP133*	*LOC111674475*	*CYP3A4*	*C8orf37-AS1*
RNASEH2B	*SOX18*	*PBRM1*	*PDB4*	*TRB*	*ITGA4*	*LINC01804*
TNFSF15	*CCHCR1*	*FMR1*	*SOX11*	*SPTAN1*	*STAR*	*CORIN*
BIRC5	*INO80*	*FAH*	*GSTM3*	*DIS3L2*	*F11*	*ZNF618*

NUP205	*ELOVL2*	*MECOM*	*GALNS*	*FLI1*	*CSTB*	*THEM4*
RAC1	*SARS2*	*IRF1*	*NUP160*	*SORL1*	*TNFRSF13B*	*NTAN1*
GATA6	*SLC47A1*	*PACRG*	*PRODH*	*ALDH2*	*UBD*	*DACH1*
MMP8	*PTPRN2*	*NOS1*	*CYP24A1*	*ETS1*	*PHKG2*	*TMEM229B*
TAP2	*SHBG*	*MIR181A1*	*ALK*	*LOC110806263*	*TMEM138*	*AHR*
MTR	*SLC13A3*	*MAOA*	*EIF2AK4*	*TPP1*	*KIRREL2*	*ALMS1P1*
ITPR1	*TFDP2*	*DYNC2I1*	*BLM*	*STK11*	*PLXDC2*	*STOX1*
PRKG1	*TFPI*	*DNAH8*	*SF3B1*	*FBN2*	*FBXL20*	*DOK6*
MIR214	*C9*	*MUC5AC*	*ECM1*	*PAX4*	*SLC22A16*	*SLC16A9*
NEUROD1	*MYRF*	*ERCC1*	*ZNF687*	*TAP1*	*DNAH17*	*PDXDC1*
RAD21	*TMEM258*	*CPLANE1*	*CADM3*	*MBP*	*KYNU*	*SYNE2*
AFG3L2	*CERS4*	*AOC3*	*INAVA*	*BCL6*	*SELENBP1*	*DNAJC16*
DYNC2I2	*PIP5K1B*	*IL19*	*IL37*	*MIR182*	*NAT8*	*GRIP1*
SFRP4	*SHROOM3*	*MT2A*	*HOXD10*	*DDC*	*DLGAP2*	*GABPA*
PGAM2	*SLC22A1*	*CDK20*	*FARSB*	*TMEM126B*	*UBE2Q2*	*SH2B1*
CHCHD2	*SLC22A2*	*GCKR*	*SGO1*	*IRGM*	*MMP20*	*AOC1*
ARPC1B	*CPS1*	*CLDN19*	*POGLUT1*	*SLC17A1*	*FADS2*	*OTOGL*
PRSS2	*WDR37*	*DAB2*	*GYG1*	*GFI1*	*RAPGEF5*	*SLC13A5*
JPH1	*WDR72*	*BCAS3*	*GNB4*	*NOTCH2NLC*	*RHD*	*ACADL*
PMP2	*PDE4D*	*MIR1908*	*PPM1K*	*ACADS*	*RHCE*	*IGF2R*
ZFAT	*SGK1*	*LINC01723*	*PDE8B*	*ASPG*	*FOXD3*	
SLURP1	*SLC6A13*	*DHX16*	*SAR1B*	*FADS1*	*TNNI3K*	

Table 3. target genes of CKD and SAA.

**80 Intersecting Genes**			
ABCB1	*P08183*	*IGF1R*	*P08069*
ACE	*P12821*	*KDR*	*P35968*
ACHE	*P22303*	*LCN2*	*P80188*
ADAM17	*P78536*	*LPA*	*P08519*
ADH1C	*P00326*	*LTF*	*P02788*
AGXT	*P21549*	*MAPK1*	*P28482*
AIFM1	*O95831*	*MAPK14*	*Q16539*
AKT1	*P31749*	*MAPK8*	*P45983*
ALB	*P02768*	*MET*	*P08581*
ANXA5	*P08758*	*MIF*	*P14174*
AR	*P10275*	*MMP1*	*P03956*
ERK	*P29323*	*MMP12*	*P39900*
BCHE	*P06276*	*MMP2*	*P08253*
BMP7	*P18075*	*MMP3*	*P08254*
CASP3	*P42574*	*MMP8*	*P22894*
CFB	*P00751*	*MMP9*	*P14780*
CREB1	*P16220*	*MTOR*	*P42345*

CTNNB1	*P35222*	*MYC*	*P01106*
CTSB	*P07858*	*NOS3*	*P29474*
CYP2C9	*P11712*	*NR3C2*	*P08235*
DAB2	*P98082*	*PAH*	*P00439*
DPP4	*P27487*	*PDE4D*	*Q08499*
EDN1	*P05305*	*PLAU*	*P00749*
EGFR	*P00533*	*PPARA*	*Q07869*
ELANE	*P08246*	*PPARG*	*P37231*
ESR1	*P03372*	*SMAD2*	*Q6SZW1*
F2	*P00734*	*RBP4*	*P02753*
F7	*P08709*	*SELP*	*P16109*
FGFR1	*P11362*	*SERPINE1*	*P05121*
FGFR2	*P21802*	*SHBG*	*P04278*
GATM	*P50440*	*SMAD3*	*P84022*
GC	*P02774*	*SRC*	*P12931*
GBA	*P04062*	*SYK*	*P43405*
GSK3B	*P49841*	*TGFB1*	*P01137*
GSR	*P00390*	*TGFBR1*	*P36897*
GSTP1	*P09211*	*TGFBR2*	*P37173*
HDAC8	*Q9BY41*	*TTR*	*P02766*
HMGCR	*P04035*	*TYMP*	*P19971*
HSP90AA1	*P07900*	*VCAM1*	*P19320*
YARS1	*P54577*	*CTSD*	*P07339*
